# Development of a radiomics and clinical feature-based nomogram for preoperative prediction of pathological grade in bladder cancer

**DOI:** 10.3389/fonc.2025.1661979

**Published:** 2025-08-27

**Authors:** Qi Zhou, Lu Ma, Yanhang Yu, Chuanao Zhang, Jun Ouyang, Caiping Mao, Zhiyu Zhang

**Affiliations:** ^1^ Department of Urology, The First Affiliated Hospital of Soochow University, Suzhou, China; ^2^ Department of Reproductive Medicine Center, The First Affiliated Hospital of Soochow University, Suzhou, China; ^3^ Department of Urology, Tongren Hospital, Shanghai Jiao Tong University School of Medicine, Shanghai, China

**Keywords:** bladder urothelial carcinoma, pathological grade, radiomics, CT texture analysis, nomogram

## Abstract

**Introduction:**

This study aimed to develop a preoperative predictive model for pathological grading of bladder urothelial carcinoma by integrating multi-parameter, thin-slice enhanced computed tomography (CT) texture features with relevant clinical indicators.

**Methods:**

CT images and clinical data were retrospectively collected from 372 individuals diagnosed with bladder urothelial carcinoma at our institution between January 2015 and October 2020. The cohort was categorized into high-grade urothelial carcinoma (HGUC; n = 190) and low-grade urothelial carcinoma (LGUC; n = 182). Participants were randomly assigned to a training group (n = 259) and a validation group (n = 113) in a 7:3 ratio. Regions of interest (ROIs) were delineated on all enhanced CT images using 3D-Slicer software, and 1,223 texture features encompassing first-order, second-order, high-order, and filtered attributes were extracted. Features with an intraclass correlation coefficient (ICC) above 0.75 were retained for further analysis via least absolute shrinkage and selection operator (LASSO) regression. A logistic regression model was constructed based on the selected features to develop a clinical prediction tool. The model’s performance was evaluated using the concordance index (C-index), calibration curve, receiver operating characteristic (ROC) curve, and decision curve analysis (DCA).

**Results:**

Eleven radiomics features demonstrated significant associations with the pathological grade of bladder urothelial carcinoma. Among the models evaluated, the logistic regression model exhibited the highest discriminative power, with an area under the curve (AUC) of 0.858. Multivariate analysis identified age and proteinuria as independent predictors. The integrated model, incorporating both clinical and imaging features, outperformed models based on clinical or radiomic data alone (AUC = 0.864).

**Conclusion:**

This study presents the first CT-based nomogram that integrates multiparametric radiomic features with comprehensive clinical indicators to preoperatively predict pathological grade in bladder urothelial carcinoma. The model offers a robust, accurate, and non-invasive tool that can facilitate individualized treatment planning and enhance clinical decision-making.

## 1 Introduction

Bladder cancer (BCa), which predominantly comprises urothelial carcinoma, is a common and potentially lethal malignancy of the urinary system (1, 2). Despite advances in therapeutic strategies, the overall prognosis for individuals with BCa remains poor (3). Accurate preoperative pathological grading is essential for selecting appropriate treatment modalities, as high-grade urothelial carcinoma is associated with a higher risk of muscle invasion and recurrence (4, 5). Current diagnostic classifications depend on invasive cystoscopic biopsy, which poses complications such as infection and discomfort (6). Therefore, there is an urgent clinical need for a non-invasive approach to predict pathological grade and inform personalized treatment planning.

Consistent with the 2025 European Association of Urology (EAU) guidelines, multiphasic computed tomography (CT) urography and, when indicated, magnetic resonance imaging (MRI) are recommended for pre-operative staging and risk stratification of bladder cancer; nevertheless, these modalities still provide only limited insight into tumor micro-architecture and molecular heterogeneity (7, 8). Recently, radiomics, a method involving the extraction of high-dimensional quantitative features from medical images, has emerged as a powerful tool in oncology for diagnosis, prognosis, and treatment response assessment (9, 10). When integrated with machine learning algorithms, radiomics has shown promise in tumor characterization and clinical decision support (11). To date, most radiomics models for predicting pathological grading in bladder cancer have relied on MRI, (12–14) while CT has been more commonly used to evaluate the risk of muscle-invasive disease (15). However, in China, CT is more accessible and cost-effective than MRI, offering faster acquisition and superior lesion visualization (16). Given these advantages, this study proposes a novel, non-invasive, quantitative prediction model based on multiparametric thin-slice enhanced CT and texture analysis. The model leverages machine learning algorithms to estimate pathological grade in individuals with bladder cancer. Additionally, we aim to construct a nomogram that integrates radiomic and clinical features to further support individualized clinical decision-making. Therefore, the objective of this retrospective study was to develop and validate a machine-learning-based nomogram that integrates radiomic features from enhanced CT with relevant clinical parameters to non-invasively predict pathological grade in individuals with bladder urothelial carcinoma.

## 2 Materials and methods

### 2.1 Patient cohort and grouping methodology

**Figure 1:**
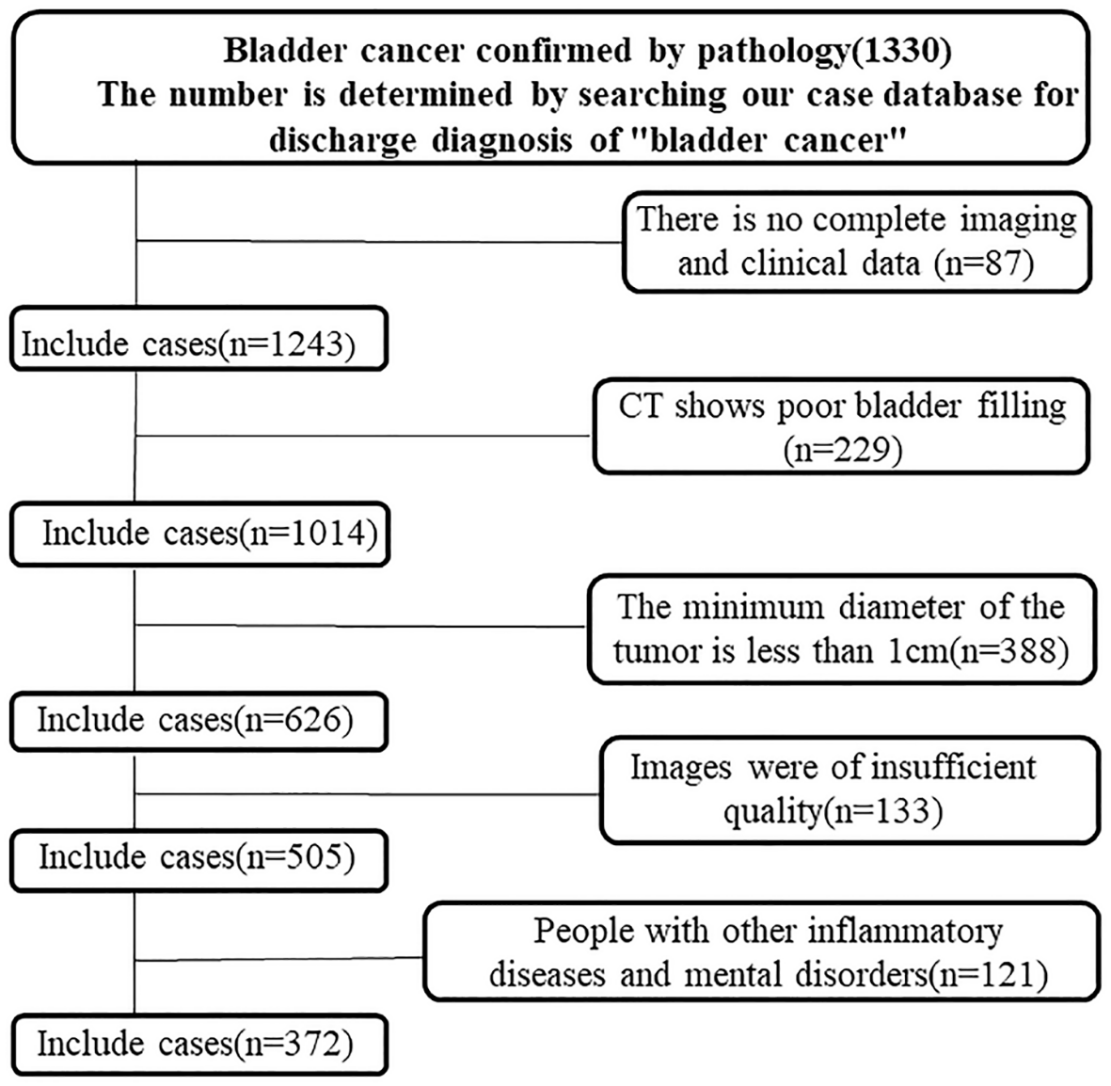
Flowchart of the enrolled patients. A total of 372 patients with bladder cancer who met the inclusion criteria were identified through the hospital’s case retrieval system and the Picture Archiving and Communication System (PACS).

This study analyzed data from 372 individuals diagnosed with urothelial carcinoma at our hospital between January 2015 and October 2020. Of these, 182 had low-grade and 190 had high-grade carcinoma. Inclusion criteria required a postoperative pathological diagnosis, complete clinical records, and a contrast-enhanced CT scan showing a bladder tumor of at least 1 cm in diameter. Exclusions included prior neoadjuvant therapy, inadequate bladder filling, tumors <1 cm, organ insufficiencies, inflammatory bladder disease, psychiatric disorders, and other malignancies. Patients were split into training and validation groups in a 7:3 ratio, as shown in **Figure 1**.

### 2.2 Collection of clinical and imaging data

Collected clinical data included height, age, sex, urinary pH, weight, presence of metabolic syndrome (MS), history of hypertension, urinary tract infections, and body mass index (BMI). It also included hematuria, urinary protein, high-density lipoprotein cholesterol (HDL-C), and tumor location, number, and size (maximum diameter). Additional data included diabetes mellitus (DM), triglyceride levels (TG), pathological grade, past medical history, and family history. Imaging data comprised thin-slice contrast-enhanced CT images of the venous and arterial phases, taken within a month prior to surgery, clearly showing bladder tumors without artifacts.

### 2.3 CT scanning procedure

All participants underwent abdominal and pelvic (bladder) arteriovenous CT scans using a Siemens 64-slice spiral CT scanner. Reconstructed images were uploaded in uncompressed DICOM format to the Picture Archiving and Communication System (PACS). Arterial and venous phase thin-layer image data were then downloaded from the PACS system.

### 2.4 Delineation of image region of interest

**Figure 2:**
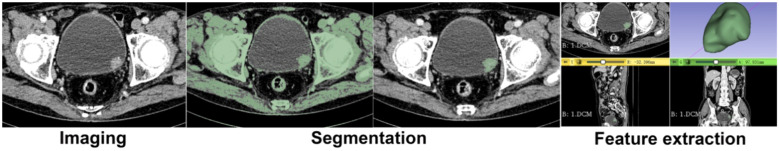
Delineation of Image Region of Interest (ROI). Semi-manual three-dimensional segmentation of the tumor, the tumor in 3D form, and the cutting of the tumor for smoothing.

Arterial and venous phase images were fused using the 3D Slicer image processing software. Two radiology residents, each with over three years of experience in urinary system imaging interpretation, independently delineated the ROI using the Threshold and Sphere Brush functions, without prior knowledge of pathological results. Semi-automatic, continuous-slice ROI delineation was performed to encompass the entire bladder tumor lesion. To ensure morphological consistency, contours were smoothed after segmentation. The ROI delineation process is illustrated in **Figure 2**. Furthermore, to encompass the entire bladder tumor as accurately as possible, the delineation line is maintained approximately 1–2 mm from the tumor’s edge. In cases of multiple foci, only the largest focus is included in the study to account for individual variability.

### 2.5 Image processing and data acquisition

Radiomic feature extraction was carried out with the Radiomics module in 3D Slicer, which calls PyRadiomics v3.0 and fully implements Image Biomarker Standardization Initiative (IBSI) definitions. To minimize inter-scanner variability, all CT volumes were (i) resampled to isotropic 1 × 1 × 1 mm³ voxels, (ii) z-score intensity-normalized, and (iii) further harmonized across scanners using the ComBat algorithm. IBSI-compliant features were then generated, including first-order statistics, shape descriptors (Shape2D, Shape3D), and higher-order texture matrices—gray-level co-occurrence (GLCM), run-length (GLRLM), size-zone (GLSZM), dependence (GLDM), and neighboring gray-tone difference (NGTDM). Multiscale information was captured with log-sigma filters (σ = 1.0, 1.5, 2.0, 2.5) and wavelet decompositions.

### 2.6 Preprocessing of texture feature data

The radiomic feature extraction process began with the use of 3D Slicer software (www.slicer.org/) to derive features from tumor image data. Using the Radiomics module, three-dimensional image features such as first-order, second-order, high-order, and filtering features were extracted (13, 14). First-order features describe global gray-level characteristics such as intensity distribution and energy. Second-order features quantify local gray-level dependencies, including autocorrelation, contrast, correlation, difference average, and difference entropy. High-order features reflect complex textural attributes, such as coarseness and heterogeneity. A total of 80 feature types were computed from each tumor ROI: first-order (18 features), 3D shape (16 features), 2D shape (14 features), gray-level co-occurrence matrix (24 features), gray-level run length matrix (16 features), gray-level size zone matrix (16 features), neighboring gray-tone difference matrix (5 features), and gray-level dependence matrix (14 features), yielding 1,223 total radiomic features. Preprocessing was performed to ensure feature quality and model compatibility. Initially, null values were imputed using the median. Features were then filtered using the intraclass correlation coefficient (ICC > 0.75) to retain those with high reproducibility. Finally, all retained features were normalized using Z-score transformation to a range of (−1, 1).

### 2.7 Extraction and screening of texture features

Effective radiomics features were selected from the training set using the Least Absolute Shrinkage and Selection Operator (LASSO) regression. Models were built using logistic regression, decision trees, support vector machines, and AdaBoost. Models were evaluated using accuracy, sensitivity, and specificity.

### 2.8 Selection and assignment of clinical factors

To develop a robust and efficient predictive model for the pathological grade of bladder cancer, diverse clinical factors were integrated. These included sex, age, height, weight, history of hypertension, urine pH, urinary tract infection, hematuria, urinary protein levels, triglycerides, BMI, diabetes, tumor location, tumor number, HDL-C, tumor size (maximum diameter), pathological grade, MS, past medical history, and family history. Given the data-intensive and complex nature of this study, categorical parameters were assigned numerical values to enhance computational efficiency and clarity in display. For instance, low-grade urothelial carcinoma was assigned a value of “0,” while other classifications were marked as “1.” For further details on the assignment of clinical factors, please refer to **Table 1**.

**Table 1:** Clinical factor assignment.

Clinical factor	Assignment”0”	Assignment”1”
Gender	Female	Male
Urinary tract infection	No	Yes
Hematuria (%)	No	Yes
Proteinuria	No	Yes
Number	Single	Multiple (>=2)
Metabolic syndrome (MS)	No	Yes
BMI	BMI<25	BMI≥25
Triglyceride	<1.7	≥1.7
High blood pressure (HBP)	No	Yes
Diabetes mellitus (DM)	No	Yes

Definition: yes = 1, no = 0.

BMI normal value = 18.5- 23.9. Triglyceride normal value = 0.45~1.69mmol/L. High-Density Lipoprotein(HDL) normal value = 0.7~2.0mmol/L. Urine pH normal value = 6.0- 6.5.

### 2.9 Construction of predictive models: comparing four algorithms

Initially, four predictive models were developed to classify the pathological grade of bladder cancer using the pre-divided training set. Accuracy, sensitivity, specificity, and the area under the receiver operating characteristic curve (AUC) were used to compare model performance. To assess the predictive power of these models, a 10-fold cross-validation procedure with 100 iterations was employed. In parallel, comparisons were made with a clinical feature-based prediction model to identify the model with superior predictive capability.

### 2.10 Statistical analysis

The software utilized in this study included 3D Slicer (version 4.10.2 for Windows 64-bit), RStudio (version 1.2.1335), and associated software packages. SPSS 22.0 (IBM) was used to analyze clinical variables, with continuous data expressed as mean ± standard deviation (x ± s). Independent samples t-tests were used to compare two groups of continuous variables, while chi-square tests were applied to categorical data. Logistic regression was used to identify independent risk factors. The effectiveness of radiological features in distinguishing between NMIBC and MIBC was evaluated using one-way analysis of variance. The LASSO regression model was implemented using the “glmnet” package, and ROC curves were plotted using the “pROC” package. Differences in AUC values between models were tested using the DeLong test, with statistical significance set at P < 0.05 (two-sided). Model performance was assessed using the concordance index (C-index), and decision curve analysis (DCA) was performed to evaluate clinical utility.

## 3 Results

The results are presented in five parts: analysis of clinical variables, imaging feature selection, construction and comparison of prediction models, performance evaluation of the integrated model, and development of a radiomic nomogram. All findings are reported based on analyses performed in the training and validation datasets.

### 3.1 Correlation analysis of clinical features

This study included 372 patients with bladder cancer. Of the participants, 301 were male and 71 were female, aged 26 to 95 years, with a mean age of 65.48 ± 12.39 years. The cohort comprised 182 individuals with low-grade urothelial carcinoma (LGUC) and 190 with high-grade urothelial carcinoma (HGUC). **Table 2** presents the clinical characteristics of the high- and low-grade groups, as well as the overall cohort. Patients were randomly assigned to a training group (n = 259) and a validation group (n = 113) in a 7:3 ratio. **Table 3** summarizes HGUC and LGUC data in both groups. Univariate analysis revealed statistically significant associations for age, urinary tract infection, hematuria, proteinuria, and tumor diameter (P < 0.05). Multivariate analysis identified age and proteinuria as independent risk factors in the clinical prediction model (P < 0.01).

**Table 2:** General information of patients in training group and verification group.

Clinical factor	LGUC (N=182)	HGUC (N=190)	U/χ2	P
Age (χ±S, year)	61.98 ± 12.29	68.85 ± 11.91	11574	<0.0001
Gender (%)			2.733	0.0983
Male	141 (77.5%)	160 (84.2%)		
Female	41 (22.5%)	30 (15.8%)		
BMI (χ±S, kg/m2)	23.56 ± 3.275	23.42 ± 3.243	17190	0.9235
Urine PH (χ±S)	6. 052 ± 0.7187	6.079 ± 0.7376	16860	0.6699
Urinary infection (%)			13.78	0.0002
Yes	81 (44.5%)	121 (63.7%)		
No	101 (55.5%)	69 (36.3%)		
Hematuria (%)			18.44	<0.0001
Yes	105 (57.7%)	149 (78.4%)		
No	77 (42.3%)	41 (21.6%)		
Proteinuria (%)			42.12	<0.0001
Yes	52 (28.6%)	118 (62.1%)		
No	130 (71.4%)	72 (37.9%)		
Tumor location (%)			6.487	0.2617
Left side wall	69 (37.9%)	86 (46.8%)		
Right side wall	49 (26.9%)	55 (28.9%)		
Bladder triangle	12 (6.6%)	9 (4.7%)		
Front wall	7 (3.8%)	11 (5.8%)		
Posterior wall	39 (21.4%)	25 (13.2%)		
Bladder vault	5 (3.4%)	4 (6%)		
Tumor number (%)			0.4112	0.5213
single	162 (89%)	165 (86.8%)		
Multiple (>=2)	20 (11%)	25 (13.2%)		
Tumor size (χ±S, mm)	19.65 ± 11.27	24.94 ± 15.05	13804	0.0007
MS (%)			0.4207	0.5166
Yes	50 (27.5%)	58 (30.5%)		
No	132 (72.5%)	132 (69.5%)		
HBP (%)			1.210	0.2714
Yes	74 (40.7%)	88 (46.3%)		
No	108 (59.3%)	102 (53.7%)		
Triglycerides (χ±S)	1.443 ± 0.8769	1.415 ± 0.8038	16390	0.3853
HDL-C (χ±S)	1.201 ± 0.3570	1.204 ± 0.3159	16933	0.7305
DM (%)			2.599	0.1070
Yes	30 (16.5%)	44 (23.2%)		
No	152 (83.5%)	146 (76.8%)		

LGUC, Low-grade urothelial carcinoma; HGUC, Low-grade urothelial carcinoma; BMI, Body mass index; MS, Metabolic syndrome; HBP, high blood pressure; HDL-C, High density lipoprotein cholesterol; DM, Diabetes mellitus.

**Table 3:** Comparison of the characteristics of HGUC and LGUC in the training group and the training group.

Clinical factor	Training group (259)		U/χ2	P	Validation group (113)		U/χ2	P
	HGUC (126)	LGUC (133)			HGUC (64)	LGUC (49)		
Age (χ±S, year)	68.83 ± 12.07	61.53 ± 12.61	5656	<0.0001	68.91 ± 11.68	63.20 ± 11.43	1046	0.002
Gender (%)			0.735	0.39			2.746	0.097
Male	103	103			57	38		
Female	23	30			7	11		
BMI (χ±S, kg/m^2^)	23.42 ± 3.37	23.28 ± 3.12	8133	0.68	23.44 ± 2.99	24.32 ± 3.58	1401	0.335
Urine PH (χ±S)	6.04 ± 0.67	6.06 ± 0.73	8368	0.98	6.14 ± 0.71	6.02 ± 0.66	1446	0.467
Urinary infection (%)			14.62	0.0001			5.470	0.019
Yes	82	55			44	23		
No	44	78			20	26		
Hematuria (%)			21.43	<0.0001			7.846	0.005
Yes	103	64			51	27		
No	23	69			13	22		
Proteinuria (%)			34.71	<0.0001			7.942	0.004
Yes	80	36			38	16		
No	46	97			26	33		
Tumor location (%)			4.755	0.446			9.543	0.089
Left side wall	59	53			27	16		
Right side wall	37	32			18	17		
Bladder triangle	7	11			2	1		
Front wall	4	7			7	0		
Posterior wall	17	26			8	13		
Bladder vault	2	4			2	2		
Tumor number (%)			0.690	0.405			3.205	0.073
single	114	116			51	45		
Multiple (>=2)	12	17			13	4		
Tumor size (χ±S, mm)	25.30 ± 15.62	18.57 ± 9.82	6320	0.0006	28.20 ± 15.99	21.76 ± 12.11	1206	0.035
MS (%)			0.302	0.582			0.094	0.758
Yes	38	36			20	14		
No	88	97			44	35		
HBP (%)			0.378	0.5385			0.0029	0.956
Yes	54	52			34	30		
No	72	81			30	27		
Triglycerides (χ±S)	1.42 ± 0.80	1.46 ± 0.97	7996	0.525	1.40 ± 0.81	1.37 ± 0.54	1455	0.513
HDL-C (χ±S)	1.21 ± 0.31	1.23 ± 0.38	8332	0.937	1.18 ± 0.31	1.11 ± 0.23	1465	0.553
DM (%)			0.709	0.399			0.430	0.511
Yes	48	44			22	14		
No	78	89			42	35		

LGUC, Low-grade urothelial carcinoma; HGUC, High-grade urothelial carcinoma; BMI, Body mass index; MS, Metabolic syndrome; HBP, High blood pressure; HDL-C, High-density lipoprotein cholesterol; DM, Diabetes mellitus.

### 3.2 Selection of imaging features

**Figure 3:**
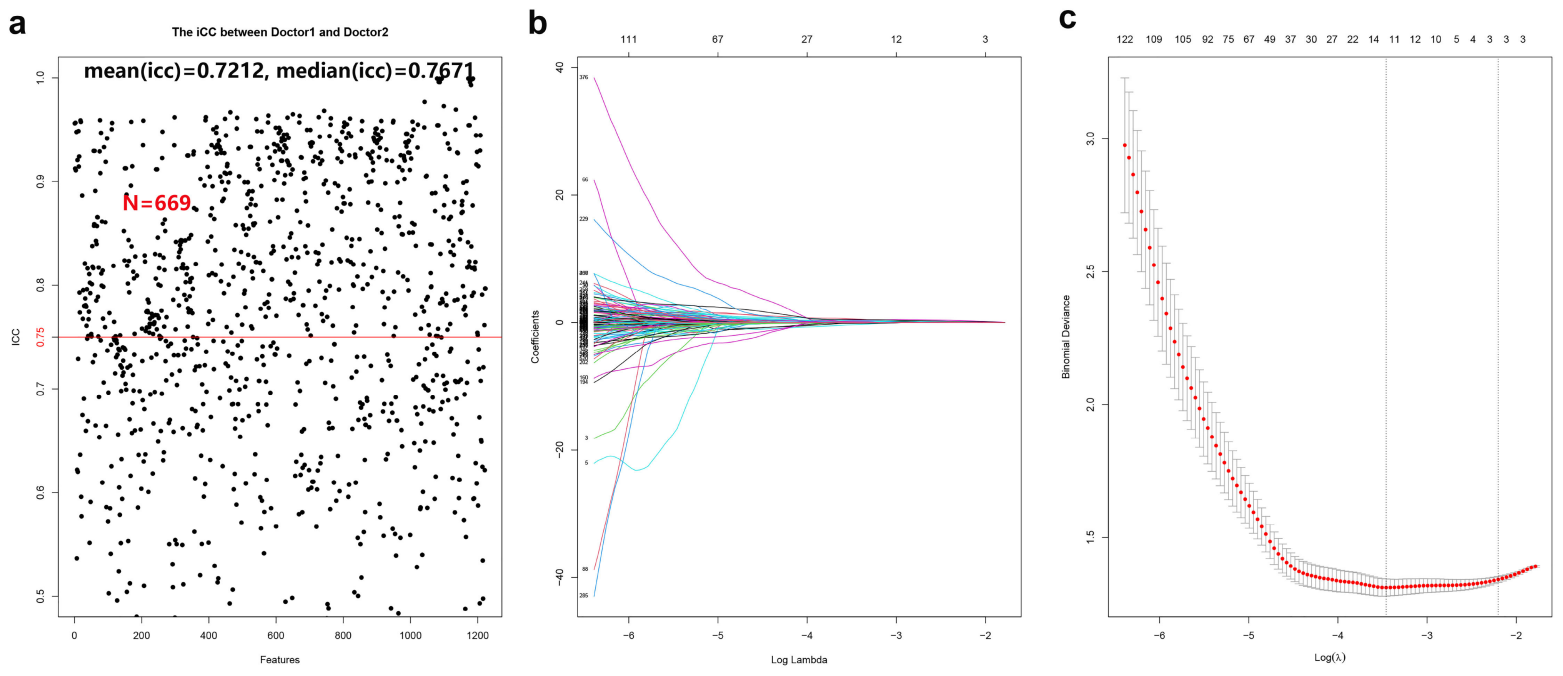
Cross-validation and feature selection for radiomic analysis. **(a)** 669 radiomic features proved to have good inter-group and intra-group consistency. **(b)** Cross-validation is used to filter the coefficient of each feature at the best logarithm (λ). As the value of λ increases, the number of features becomes less and less. **(c)** Use cross-validation to generate coefficients corresponding to the logarithmic (λ) value (minimum variance). Draw vertical lines with 11 selected radiological features.

A total of 699 radiomic features demonstrated strong inter- and intra-observer reproducibility, with ICC values of 0.75 or higher (mean ICC = 0.748; median = 0.807) (see **Figure 3a**). One-way ANOVA identified 651 features with significant differences between HGUC and LGUC (P < 0.05), which were subsequently entered into a LASSO regression model for feature selection (see **Figure 3b**). Using the minimum criterion from 10-fold cross-validation, 11 features were ultimately selected to build the predictive model (see **Figures 3b, c**). Feature selection and modeling were performed using the “glmnet” and “pROC” packages in R.

### 3.3 Model construction and evaluation

**Figure 4:**
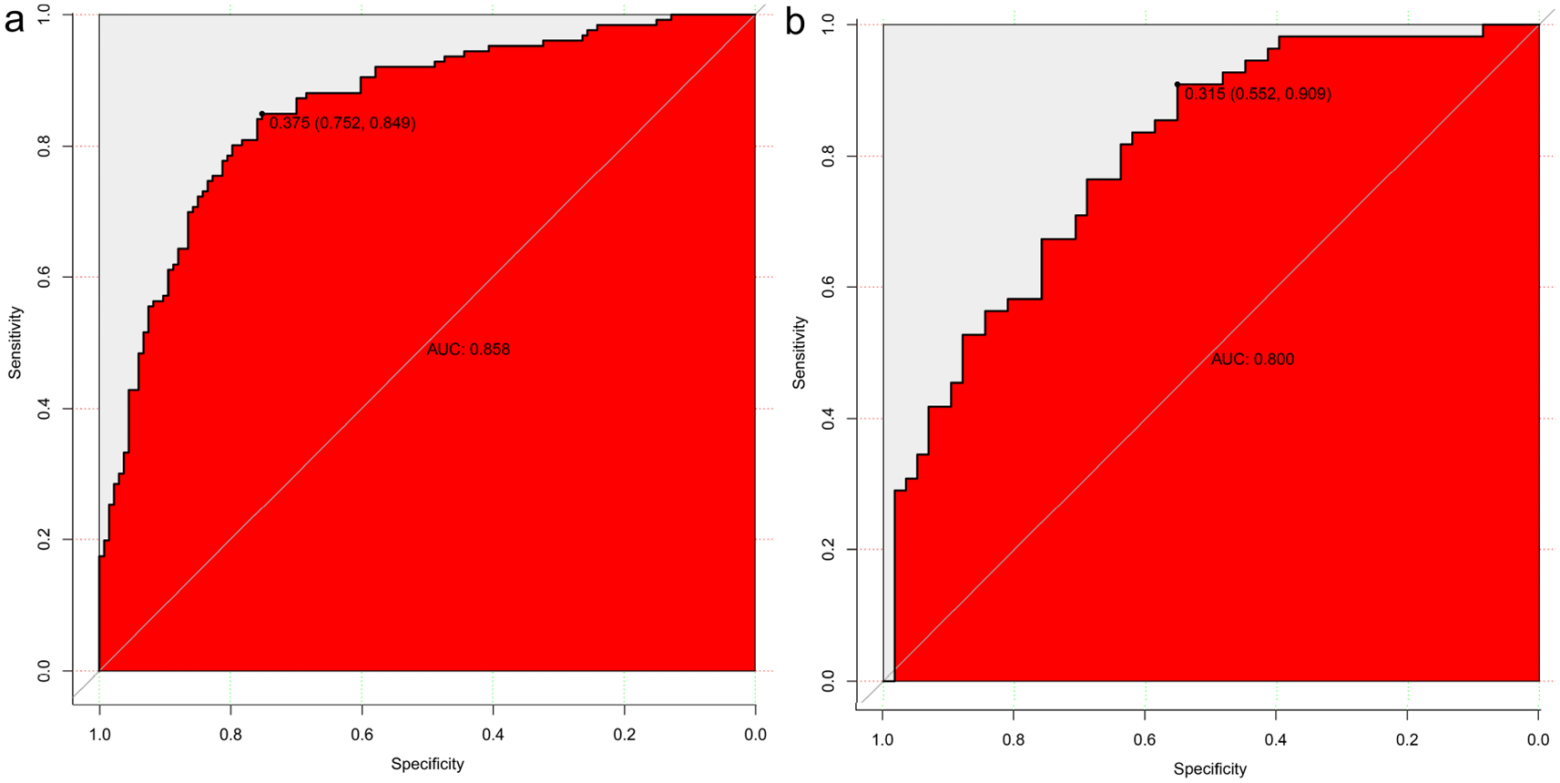
Model Construction and Evaluation. Four predictive models were constructed using the training set. Logistic regression achieved the best diagnostic performance, with area under the ROC curve (AUC) values of 0.858 in the training cohort **(a)** and 0.800 in the validation cohort **(b)**, demonstrating strong model generalizability.

Eleven radiomic features were finally selected for model construction: three first-order statistics (Entropy, Kurtosis, Skewness), five texture descriptors (GLCM_Contrast, GLCM_DifferenceVariance, GLRLM_LongRunEmphasis, GLDM_DependenceNonUniformity, NGTDM_Coarseness), and three shape indices (LongestAxisLength, SurfaceArea-to-Volume Ratio, Sphericity). Biologically, higher Entropy and Kurtosis indicate a disordered and peaked intensity distribution, often reflecting necrosis and marked cellular atypia characteristic of high-grade tumors. Texture metrics such as GLCM_Contrast and GLRLM_LongRunEmphasis quantify fine-scale grey-level fluctuations that mirror micro-architectural heterogeneity created by variable cell density, stromal content, and angiogenesis. Shape features capture macroscopic invasiveness; for example, an increased LongestAxisLength and reduced Sphericity signify elongation and irregular growth patterns commonly observed in aggressive lesions. Using these 11 features, four classifiers were evaluated—logistic regression, support vector machine (SVM), AdaBoost, and decision tree. Logistic regression showed the best overall clinical utility. In the training cohort, the AUCs were 0.858 (logistic regression), 0.872 (decision tree), 0.852 (SVM), and 0.789 (AdaBoost). For logistic regression, sensitivity, specificity, and accuracy in the training set were 75.40%, 82.71%, and 79.15%, respectively (95% CI: 0.812–0.903). In the validation set these metrics were 76.36%, 68.97%, and 72.57% (95% CI: 0.719–0.881). Thus, logistic regression maintained balanced, high diagnostic performance across both cohorts (AUC = 0.858 training; 0.800 validation; **Figure 4**). Although the AUC declined from 0.858 in the training set to 0.800 in the internal validation set, the overlapping 95% confidence intervals and well-aligned calibration curves suggest that significant overfitting did not occur. This modest reduction likely reflects normal sampling variability and underscores the importance of the forthcoming external validation study. **Table 4** summarizes the diagnostic metrics for all models.

**Table 4:** Comparison of four algorithm models based on the training group.

Algorithm models	Group	Accuracy (%)	Sensitivity (%)	Specificity (%)	95% CI	AUC
Logistic regression	Train	79.15	75.40	82.71	0.812-0.903	0.858
	Test	72.57	76.36	68.97	0.719-0.881	0.800
Decision tree	Train	83.79	80.16	87.22	0.829-0.914	0.872
	Test	73.45	74.55	72.41	0.573-0.784	0.679
SVM	Train	75.68	65.08	85.71	0.806-0.898	0.852
	Test	69.03	65.45	72.41	0.729-0.887	0.808
ADABOST	Train	78.76	84.92	72.93	0.740-0.839	0.789
	Test	69.03	85.45	53.44	0.615-0.775	0.695

SVM, Support vector machine; ADABOOST, Adaptive boosting; CI, Confidence interval; AUC, Area under the receiver operating characteristic curve.

### 3.4 Predictive model development and analysis

**Figure 5:**
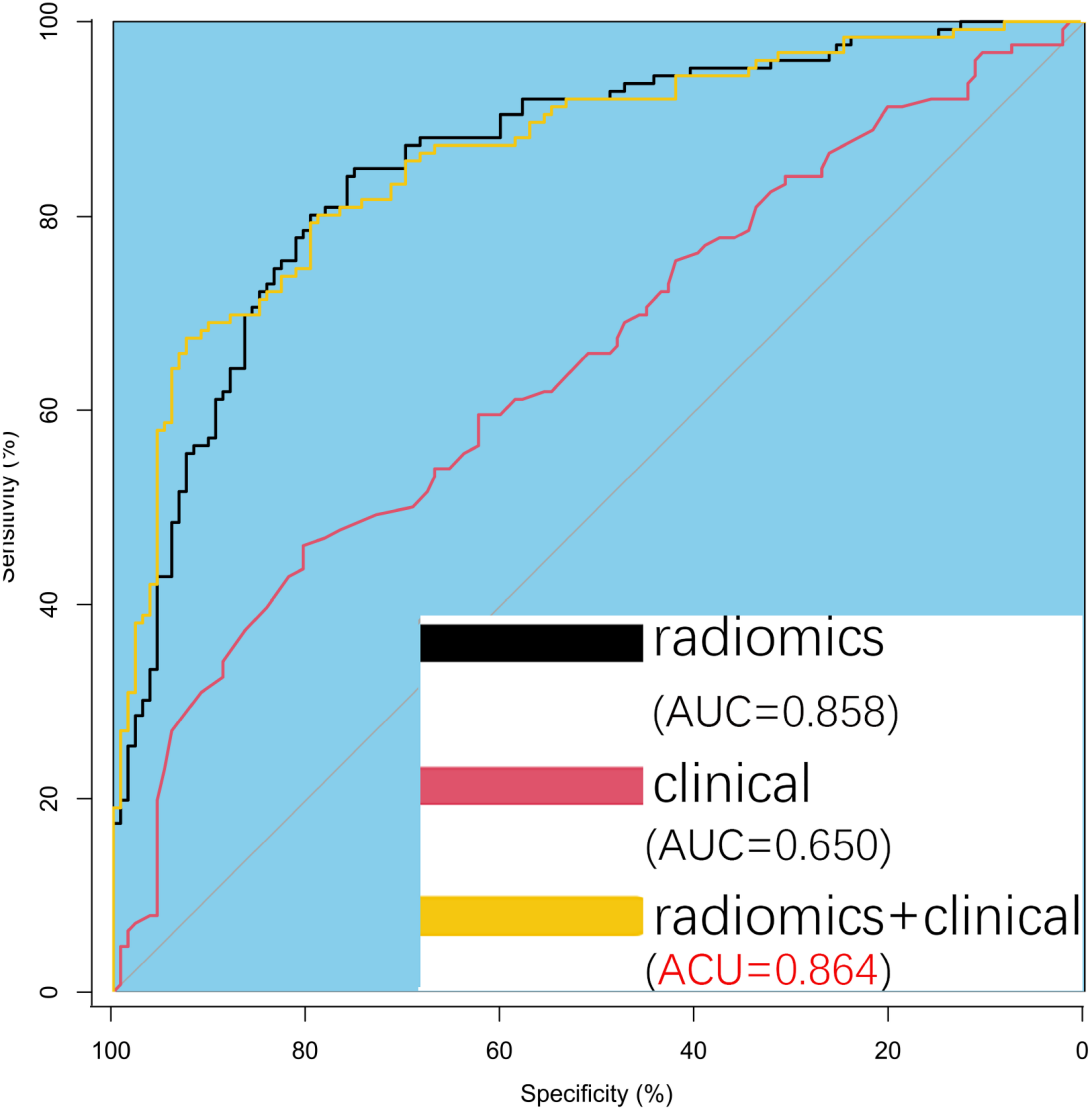
Predictive Model Development and Analysis. Receiver operating characteristic (ROC) curves comparing the three models show a significant difference between the radiomics model and the clinical model. The comprehensive model (radiomics + clinical) performs slightly better than the radiomics model alone, though the difference is minimal.

Univariate and multivariate analyses identified age and proteinuria as independent risk factors for bladder cancer pathological grade. A clinical prediction model incorporating these two variables was developed in the training group and compared with a radiomics-only model. The AUC values for the radiomics and clinical models were 0.858 and 0.650, respectively, with a statistically significant difference between them (P = 0.001). **Figure 5** presents ROC curves for the clinical, radiomics, and combined models. The radiomics model outperformed the clinical model in predicting pathological grade. A comprehensive model integrating radiomics and clinical factors achieved improved performance (AUC = 0.864).

### 3.5 Development of a radiomic nomogram with clinical integration

**Figure 6:**
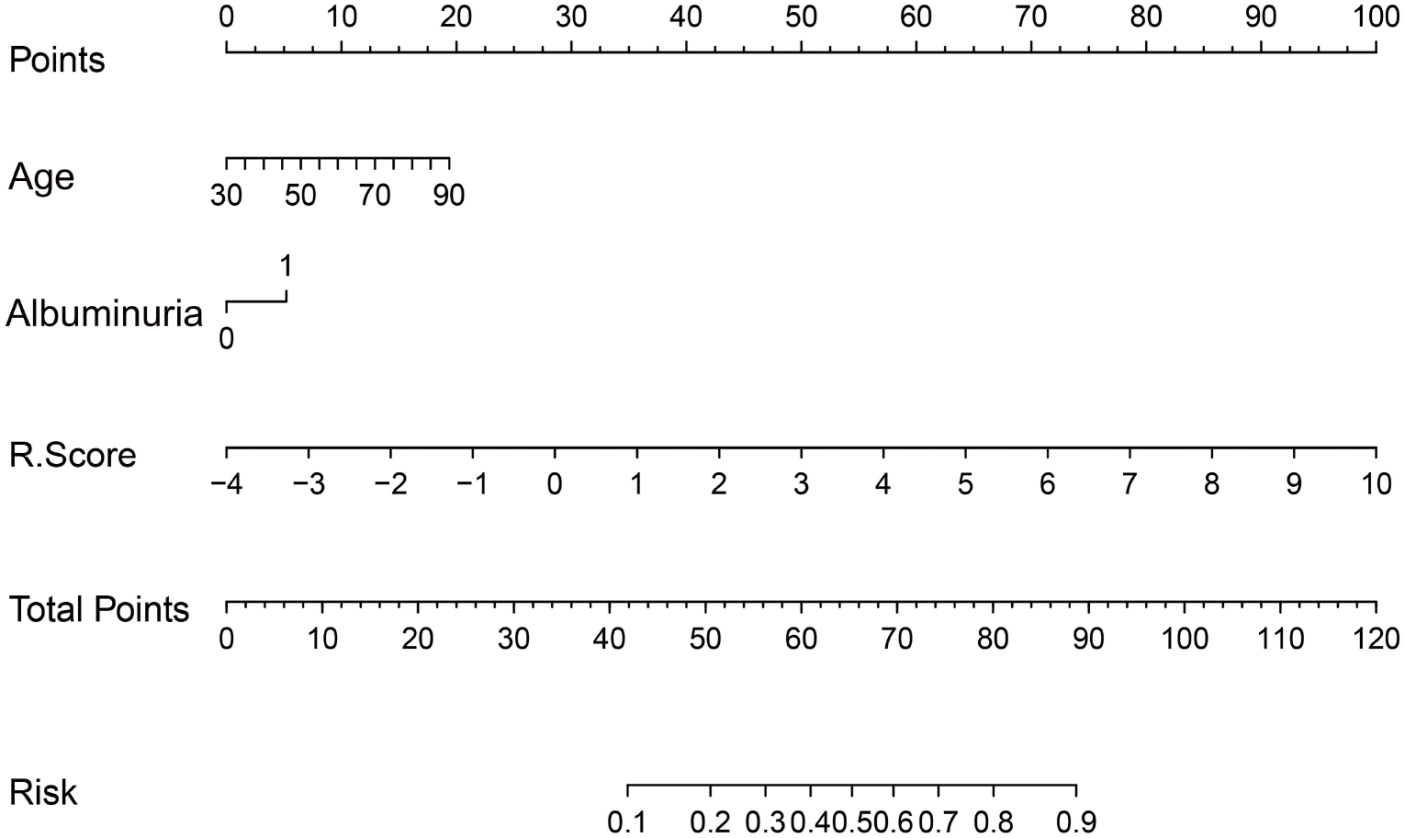
The nomogram to predict the probability of clinically high-grade urothelial carcinoma. Note: Each variable is plotted on its corresponding axis. To calculate a score, draw a vertical line from the variable value to the ‘Score’ axis. Sum the individual scores and locate the total on the ‘Total Score’ axis. Drawing a vertical line downward from this point yields the predicted probability of high-grade urothelial carcinoma.

**Figure 7:**
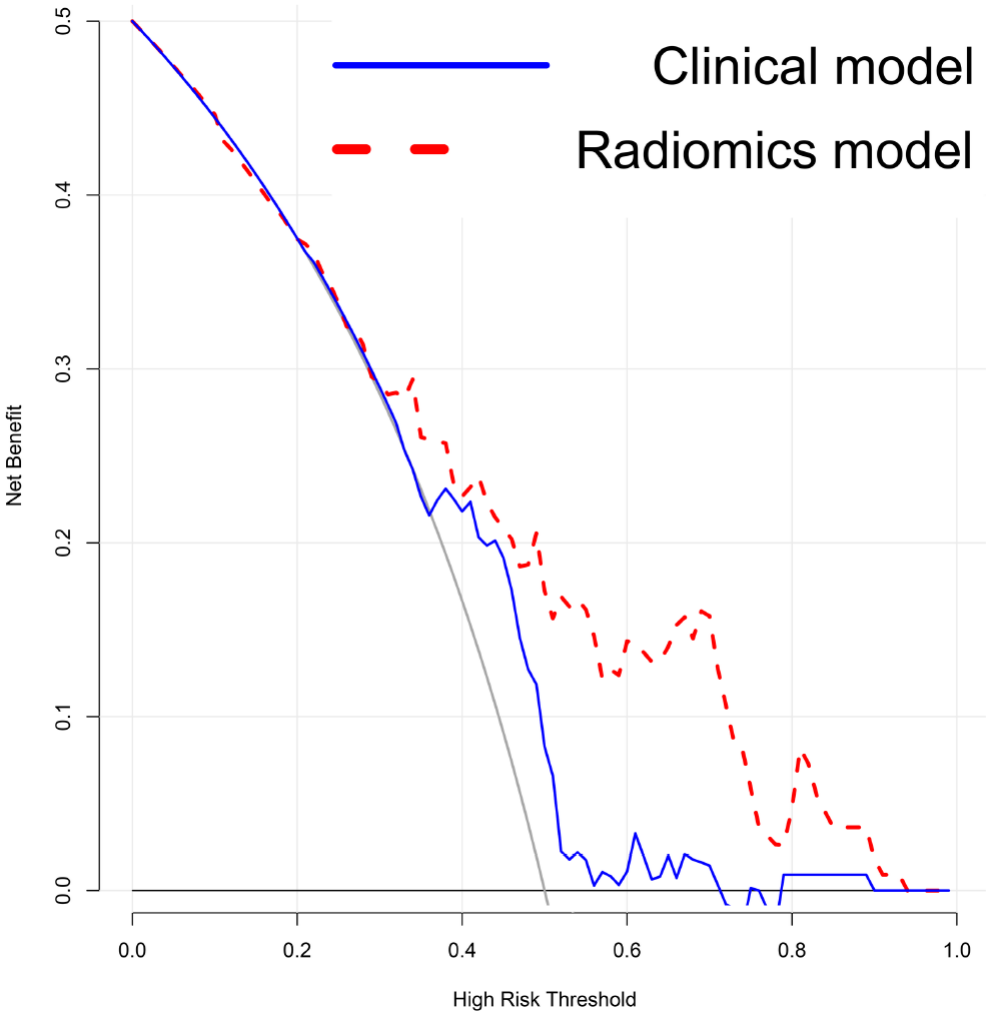
Decision Curve Analysis (DCA) of the models. The DCA shows that the combined radiomics and clinical model provides the greatest net benefit compared to the clinical-only and radiomics-only models.”.

**Figure 8:**
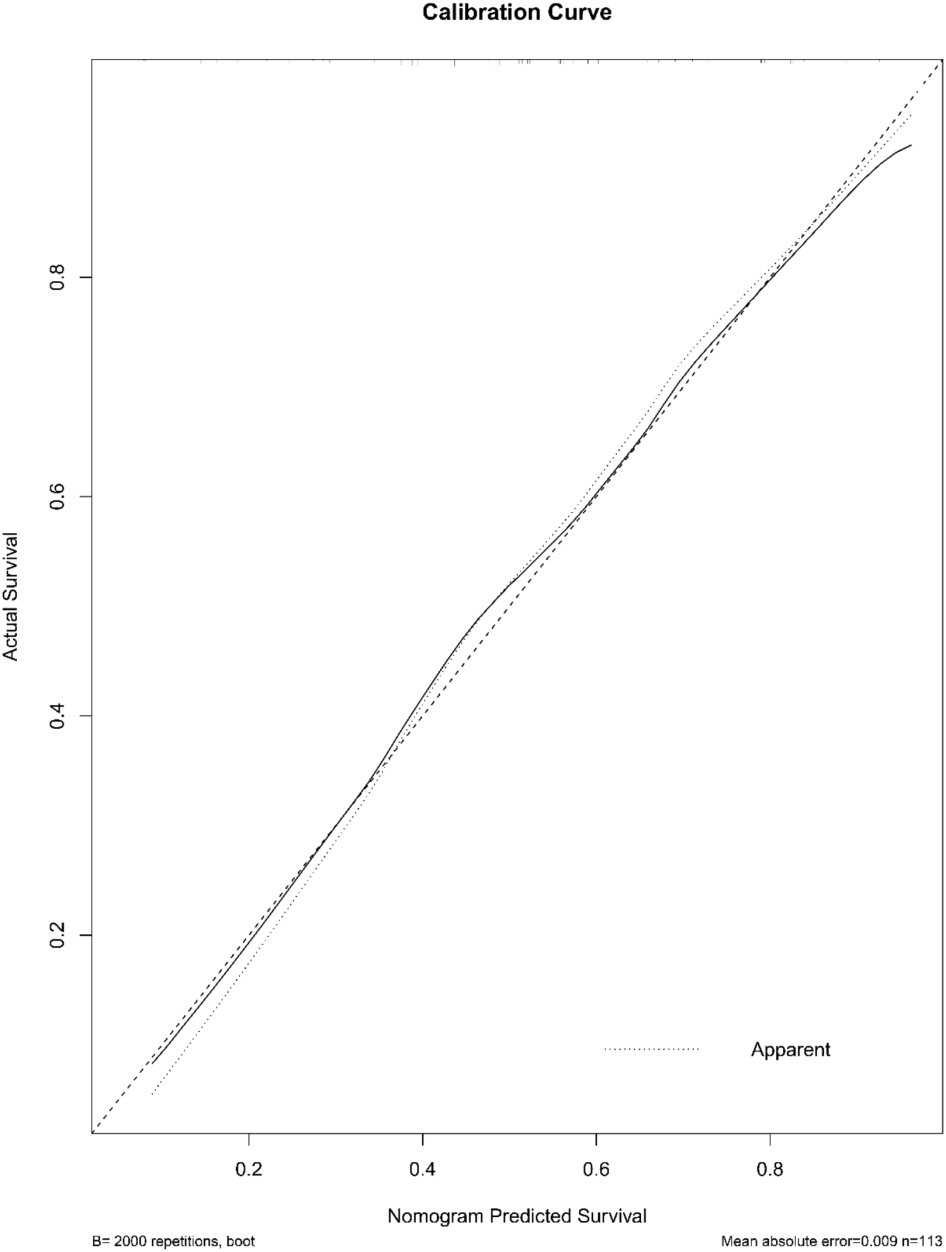
The calibration curve of the nomogram. The curve illustrates the agreement between predicted probabilities and actual outcomes for high-grade urothelial carcinoma. A 2,000-sample bootstrap was used to generate a bias-corrected curve. The 45° diagonal line represents perfect calibration.

A radiomic nomogram was developed by integrating clinical factors (age and proteinuria) with the radiomics score (Rad-Score), as shown in **Figure 6**. DCA indicated that the radiomics + clinical model demonstrated the highest net clinical benefit (**Figure 7**). The calibration curve assessed the nomogram’s goodness of fit, where the 45° dashed line represents the ideal predictive model and the solid black line reflects the nomogram’s actual performance (**Figure 8**). Closer proximity of the black line to the ideal diagonal indicates higher calibration accuracy.

To improve the clinical interpretability and predictive efficiency of the radiomics model, it was merged with the clinical model to generate a more intuitive and practical nomogram. Due to the complexity of incorporating 11 radiomic features individually, dimensionality was reduced by summarizing these features into a composite Rad-Score. The Rad-Score was calculated using the following formula:

Rad-scores: Log-sigma-1-0-mm-3D_glcm_Autocorrelation*1.03823

-Log-sigma-1-5-mm-3D_firstorder_Kurtosis*0.11197

+Log-sigma-2-0-mm-3D_glcm_JointAverage*0.12997

-Log-sigma-2-5-mm-3D_gldm_LowGrayLevelEmphasis*

-0.38671-Wavelet-LHH_glcm_Autocorrelation*1.72581

-Wavelet-LHH_glszm_SmallAreaHighGrayLevelEmphasis*0.88659

+Wavelet-LHL_gldm_LargeDependence*0.45270

+Wavelet-LLH_glcm_JointAverage*3.25086

-Original_shape_MajorAxisLength*0.05676

+Original_shape_MinorAxisLength*0.57564

+Original_shape_Maximum2DDiameterSlice*0.87998

## 4 Discussion

Bladder tumors are among the most common malignant tumors of the urinary system worldwide, with high incidence and mortality rates (1, 2). Pathological grading is a crucial prognostic indicator that guides clinical treatment decisions (4, 5). The World Health Organization (WHO) introduced a histological grading system in 1973, which was later refined into low-grade (LG) and high-grade (HG) categories in the 2004 and 2016 classifications to improve prognostic stratification (17). High-grade tumors are associated with significantly greater risks of muscle invasion and metastasis, reducing the 5-year survival rate by 30% to 50% compared with low-grade tumors (18). Therefore, tumor grade not only reflects biological aggressiveness but also informs individualized treatment strategies such as intravesical therapy or radical cystectomy (19).

However, accurate preoperative prediction of pathological grade remains challenging. Due to the spatial heterogeneity of bladder tumors, biopsy samples may not adequately represent the entire tumor, with approximately 20% of postoperative grades differing from preoperative assessments (20). Moreover, diagnostic variation among pathologists can reach 18.5% due to the subjective nature of morphological evaluation (21). Molecular studies have shown that tumors of the same pathological grade may exhibit divergent behavior, influenced by molecular markers such as FGFR3 and TP53 mutations, which are not captured by conventional grading (22, 23). These limitations may delay timely intervention for high-risk patients or lead to overtreatment of low-risk individuals.

Radiomics has recently emerged as a promising tool in precision oncology, involving the high-throughput extraction of quantitative features from medical images combined with machine learning to build predictive models (9–11). Numerous studies have demonstrated that radiomics can capture tumor heterogeneity, aiding in prognosis prediction, molecular subtyping, and treatment response assessment across various malignancies (24–26). In lung cancer, a CT-based radiomic model predicted overall survival in non-small cell lung cancer (NSCLC) patients with a concordance index (C-index) of 0.69, outperforming the traditional TNM staging system (C-index = 0.58) (24). This finding underscores radiomics’ ability to reflect intratumoral heterogeneity, supporting personalized treatment planning. In glioma research, Parmar et al. successfully differentiated IDH-mutant from wild-type tumors using MRI-based radiomics, achieving 89% concordance with genetic testing (AUC = 0.85), thereby reducing the need for invasive biopsy (25). Similarly, in breast cancer, advanced radiomics based on dynamic contrast-enhanced MRI (DCE-MRI) predicted axillary lymph node metastasis with an AUC of 0.82, providing a non-invasive tool for surgical decision-making (26). Collectively, these studies highlight radiomics’ potential to extend beyond traditional imaging by characterizing tumors at functional and molecular levels.

With advances in multiparametric MRI and CT technologies, radiomics is demonstrating strong potential in predicting the pathological grade of bladder cancer. Li et al. (12) developed a non-invasive preoperative nomogram based on a multiparametric MRI radiomics approach (T2WI, DWI, ADC maps) to distinguish low-grade from high-grade tumors in NMIBC. Their radiomics model achieved an AUC of 0.910, while a combined model incorporating clinical indicators increased performance to an AUC of 0.931. In contrast, CT-based radiomics studies have primarily focused on predicting muscle invasion in bladder cancer (13). Zhang et al. (13) reported a radiomics model derived from arterial-phase CT images that accurately predicted muscle invasion, achieving an AUC of 0.89, with a sensitivity of 84.3% and a specificity of 81.9%. Similarly, another study developed and validated a CT-based radiomics model to predict muscle invasion in bladder cancer, demonstrating good diagnostic performance with AUCs of 0.885 (training), 0.820 (internal testing), and 0.784 (external testing), suggesting its potential for preoperative evaluation (27).

Few studies have investigated CT-based radiomics for predicting pathological grade in bladder cancer. While some studies, such as Zhang et al. (28), have explored CT-based radiomics for this purpose, they often focus solely on imaging features without fully leveraging the potential of integrated clinical information. In our study, we developed a predictive model by integrating multiparametric thin-slice CT-derived radiomics features with clinical indicators such as age and hematuria severity. The combined model achieved an AUC of 0.864, significantly outperforming the radiomics-only model (AUC = 0.858, p = 0.012) and the clinical-only model (AUC = 0.650, p < 0.001). The model’s overall predictive accuracy reached 79.15%, likely driven by two complementary mechanisms: First, advanced radiomic descriptors capture spatial heterogeneity and invasive morphology: high-order texture metrics such as GLCM_Contrast and GLDM_DependenceNonUniformity, together with first-order Kurtosis, quantify the abrupt grey-level transitions produced by intermixed necrotic, haemorrhagic, and highly cellular regions, while shape indices showing reduced sphericity and increased major-axis length recreate the irregular, finger-like invasion characteristic of aggressive tumors. Second, the incorporated clinical indicators—most notably persistent macroscopic haematuria—directly reflect biological aggressiveness through their association with ongoing mucosal injury and an inflammatory micro-environment. By harmonizing these radiomic and clinical signatures, the model integrates micro-architectural complexity with patient-level disease activity, thereby achieving robust discrimination between low- and high-grade lesions. By integrating multidimensional clinical and imaging data, this model improves the accuracy of preoperative grading (AUC=0.864 vs. standalone imaging model AUC=0.858) and may help identify hidden high-grade components, thereby supporting individualized treatment decisions, such as the selection of candidates for neoadjuvant chemotherapy.

Meanwhile, the decision curve analysis indicates that our nomogram could provide meaningful clinical benefit by improving individualized risk assessment for patients with bladder cancer. Integrated into the clinical workflow, the nomogram may assist clinicians in identifying high-risk patients who could benefit from more aggressive management, thereby supporting more precise and personalized treatment decisions.

In this study, we developed a combined preoperative pathological grading prediction model for bladder cancer by integrating multiparametric CT-based radiomics features with key clinical indicators. This comprehensive model significantly outperformed single-source models, providing a non-invasive and multidimensional tool to inform personalized treatment decisions. However, this study has some limitations. First, the retrospective design introduces potential selection bias, and reliance on single-center data may limit generalizability. Although internal validation confirmed model stability (cross-validation AUC = 0.864), an independent, multi-center validation study is currently being planned to confirm the nomogram’s robustness across different institutions and imaging protocols. Second, radiomics feature extraction depends heavily on image preprocessing steps such as registration and normalization; differences in acquisition parameters across institutions may affect feature reproducibility. Standardizing image acquisition and processing workflows should be prioritized in future research. Lastly, the model does not incorporate molecular biomarkers, despite evidence that molecular alterations may complement imaging and clinical data to improve the detection of occult high-grade components (29). Future work should involve prospective multicenter studies that integrate molecular multi-omics data, enabling the development of a comprehensive imaging-clinical-molecular fusion model to support a “holographic” assessment system for bladder cancer grading. This would enhance precision oncology by making predictions more interpretable, actionable, and broadly applicable. With further validation and refinement, this model has the potential to support more precise, individualized treatment decisions in bladder cancer management.

## Data Availability

The datasets presented in this study can be found in online repositories. The names of the repository/repositories and accession number(s) can be found below: https://figshare.com/s/1c47062e7922c5eea0f1.
